# Modeling of Cure Kinetics and Rheological Behavior of an Epoxy Resin Using DSC and Rheometry

**DOI:** 10.3390/molecules31040640

**Published:** 2026-02-12

**Authors:** Xueqin Yang, Haijun Chen, Yamei Wang, Wenjian Zheng, Jie Sun, Yaodong Liu, Jintang Zhou

**Affiliations:** 1 Suzhou Laboratory, Suzhou 215123, China; yangxq01@szlab.ac.cn (X.Y.);; 2 College of Materials Science and Technology, Nanjing University of Aeronautics and Astronautics, Nanjing 211100, China

**Keywords:** epoxy resin, rheology, cure kinetics, DSC, rheological model

## Abstract

Epoxy resins with excellent overall performance, are widely used in aerospace, automotive, and related fields, frequently in combination with reinforcing fibers to fabricate composites. To enable controllable epoxy processing for prepreg fabrication and composite forming, a rheological model and a curing kinetics model were developed and experimentally validated for an epoxy resin. Rotational rheometry was conducted to quantify the viscosity evolution with temperature and time, enabling construction of a corresponding rheological model. Comparison between model predictions and experimental measurements exhibited a high level of consistency across a wide temperature range. Furthermore, differential scanning calorimetry (DSC) was employed to measure heat-flow curves at different heating rates. The degree of curing was calculated from the heat-flow data, and an autocatalytic curing kinetics model was established based on a reaction kinetics formulation. And the accuracy of the model was verified by isothermal experiments. The developed rheological model provides a theoretical basis and practical guidance for resin processing and prepreg fabrication, whereas the curing kinetics model supports the design and control of curing and forming schedules for epoxy-matrix composites.

## 1. Introduction

Epoxy resins are widely used as matrices for high-performance fiber-reinforced composites in aerospace, automobiles, transportation and related engineering fields due to their excellent mechanical properties, chemical resistance, and processability [1,2,3,4,5]. In practical manufacturing, epoxy-based prepregs and composite parts are typically produced through a sequence of heating, impregnation, curing and forming steps. The quality and consistency of these products are strongly governed by the evolution of resin viscosity and the progression of the curing reaction, which together determine key processing outcomes such as fiber impregnation, resin flow and bleed, void removal, and final degree of cure [6,7]. Consequently, establishing predictive models for rheological evolution and cure kinetics are essential for defining robust processing windows and designing reliable curing schedules. Such models not only deepen the understanding of curing behavior and underlying reaction mechanisms, but also provide a scientific basis for optimizing processing parameters in resin-based composite manufacturing [8,9]. Although chemorheological models and cure kinetics have been reported for thermosetting systems, their predictive accuracy is often insufficiently validated, particularly under isothermal conditions [7,10]. Consequently, practical processing design and composite forming remain constrained.

Resin rheology plays a direct and decisive role in epoxy processing and prepreg fabrication [7,11,12]. The viscosity–temperature response governs resin flow, fiber wetting, and impregnation, whereas the time-dependent viscosity increase during curing progressively restricts flow and ultimately limits the processing time [7,13]. Rotational rheometry provides an effective route to quantify viscosity–temperature behavior under programmed heating as well as viscosity evolution under isothermal conditions. Based on these datasets, chemorheological models can be established to describe viscosity development within the processing-relevant temperature window, enabling quantitative definition of temperature ranges and time constraints for manufacturing [13,14]. Among the available formulations, the double Arrhenius viscosity model is extensively applied in empirical approaches and is recognized for its broad applicability [15,16].

Differential scanning calorimetry (DSC) has been widely employed to characterize the non-isothermal curing behavior of thermosetting resins and to extract kinetic parameters from heat-flow responses acquired at different heating rates [7,17,18,19]. In kinetic analyses, the *n*th-order model and the autocatalytic model are among the most commonly used formulations [20,21]. The *n*th-order model is often adopted as a baseline because of its mathematical simplicity. However, some epoxy systems exhibit pronounced autocatalytic features due to the generation of reactive species during curing [17,20,22]. In such cases, a single *n*th-order description may not adequately capture the curing behavior over the full conversion range, and an autocatalytic formulation is therefore more appropriate. Moreover, beyond non-isothermal fitting, validation against isothermal curing data is required to ensure that the identified kinetic model is reliable for processing design [10,18].

Previous studies have investigated rheological and curing kinetics models for resins. However, systematic validation of the accuracy of these models under non-isothermal and/or isothermal conditions remains limited. In this study, the rheological behavior and curing kinetics of a high-performance epoxy resin were systematically investigated with the aim of providing modeling tools for prepreg processing and composite curing. Viscosity–temperature profiles and isothermal viscosity evolutions were measured using rotational rheometry, and a rheological model was established and validated by comparison with experimental data. The non-isothermal curing behavior was characterized by DSC at multiple heating rates. An *n*th-order kinetic model was first evaluated based on the DSC-derived conversion data, and its applicability to the resin system was assessed. The autocatalytic nature of the curing reaction was then examined, and a two-parameter autocatalytic kinetic model was developed to describe the curing process. The model was validated using both non-isothermal and isothermal datasets, demonstrating good agreement between predictions and experiments. The resulting rheological and kinetic models provide a quantitative basis for the processing-window definition and curing-schedule design for the epoxy resin and its composite systems.

## 2. Results and Discussion

### 2.1. Rheological Equations of Epoxy Resin

**Figure 1:**
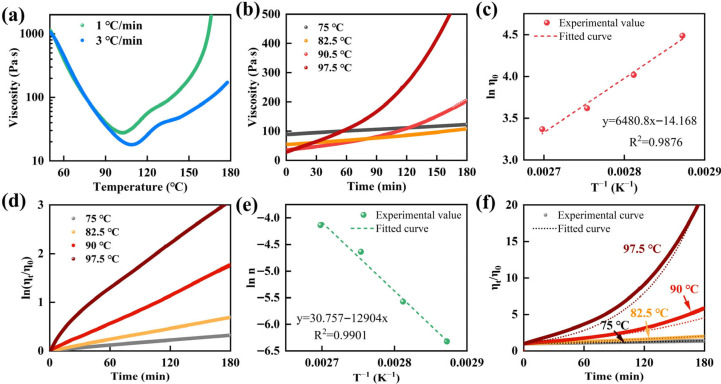
(**a**) Viscosity–temperature curves of the epoxy resin. (**b**) Viscosity–time curves of the epoxy resin under different constant temperatures. (**c**) Experimental values and fitted curves for $\ln\eta_{0}$ versus 1/*T*. (**d**) The curves of $\ln\left(\eta_{t}/\eta_{0}\right)$ versus time under different constant temperature. (**e**) Experimental values and fitted curves for lnn versus 1/*T*. (**f**) Comparison of experimental data with fitted curves for the viscosity equation.

Chemical rheological models are widely used to describe and interpret the rheological evolution of thermosetting resins during curing, thereby providing guidance for resin processing and prepreg fabrication [8,13,23]. Accordingly, the viscosity–temperature behavior of the epoxy resin was characterized, as shown in Figure 1. Figure 1a presented the viscosity–temperature profiles measured at heating rates of 1 and 3 °C/min over the range of 50–180 °C. The viscosity decreased sharply with increasing temperature at the early stage, which was attributed to enhanced segmental mobility and increased flowability of the polymer chains [24,25,26]. With further heating, the viscosity started to increase at approximately 100 °C and then rose progressively. This trend suggested the onset and gradual development of a cross-linked three-dimensional network, which increasingly constrained chain-segment mobility and consequently led to a pronounced increase in the resin viscosity. Upon heating above 100 °C, the curing reaction progressively initiated, resulting in a concomitant increase in resin viscosity. By contrast, the viscosity remained below 100 Pa·s throughout the 75–100 °C interval in accordance with the requirements of the prepreg process. On this basis, 75 °C, 82.5 °C, 90.5 °C and 97.5 °C were selected as the isothermal viscosities to characterize the rheological behavior. As shown in Figure 1b, the epoxy resin exhibited a gradual time-dependent increase in viscosity during isothermal holding.

Within the full processing temperature window, the viscosity ($\eta_{t}$) of the resin at time *t* conforms to the double Arrhenius viscosity model [15,16,27] referenced to the initial viscosity ($\eta_{0}$) of the epoxy resin, as follows:

(1) $$\eta_{t}/\eta_{0}=exp(nt)$$

where n is the model parameter and *t* is the time. $\eta_{0}$ and n conform to the double Arrhenius viscosity equation, that is:

(2) $$\eta_{0}=k_{1}exp(k_{2}/T)$$

(3) $$n=k_{3}exp(k_{4}/T)$$

where $k_{1}$, $k_{2}$, $k_{3}$, and $k_{4}$ are the parameters of the chemorheological model of thermosetting resin and *T* is the temperature.

To examine the temperature (*T*) dependence of the initial viscosity ($\eta_{0}$), Equation (2) was transformed as

(4) $$\ln\eta_{0}=\ln k_{1}+k_{2}/T$$

Furthermore, the viscosity–time curves of the epoxy resin at 75 °C, 82.5 °C, 90.5 °C and 97.5 °C were further analyzed to extract the corresponding initial viscosity ln${\;\eta }_{0}$ at each temperature. By plotting the relationship between $\ln\eta_{0}$ and 1/*T*, a linear relationship between $\ln\eta_{0}$ and 1/*T* was established, as shown in Figure 1c. Linear regression of ln${\;\eta }_{0}$ versus 1/*T* was performed, and the fitted intercept and slope were determined to be *lnk_1_* = −14.168 and *k_2_* = 6480.8, respectively. Therefore, the value of *k_1_* was 7.029 × 10^−7^. Consequently, the relationship between *η_0_* and *T* for epoxy resin based on Equation (2) was expressed as

(5) $$\eta_{0}=7.029\times 10^{-7}exp(6480.8/T)$$

To obtain the relationship between *n* and *T*, Equation (3) was transformed as follows:

(6) $$\ln n=\ln k_{3}+k_{4}/T$$

And Equation (1) was converted into

(7) $$\ln\left(\eta_{t}/\eta_{0}\right)=nt$$

Based on Equation (7), $\ln\;(\eta_{t}/\eta_{0})$ was plotted as a function of time (*t*) at the selected temperature in Figure 1d. The resulting datasets were fitted to extract the parameter *n* at each temperature, and the obtained *n* values were summarized in Table 1. To ensure sufficient numerical accuracy in modeling, the fitted parameters were reported with extended decimal places. However, their physical reliability was limited by experimental uncertainty and fitting sensitivity, and minor numerical variations should not be overinterpreted.

Based on the *n* values given in Table 1, these values were transformed by the natural logarithm, and then linear fitting was performed with 1/*T* as the independent variable. The results were shown in Figure 1e. Based on the above analysis, the fitted slope and intercept were *k_4_* = −12,904 and *lnk_3_* = 30.757, respectively. Therefore, based on Equation (6), the functional relationship between *ln n* and *T* was established as follows:

(8) lnn=30.757−12904/T

By incorporating Equation (8) into Equation (1), Equation (9) was derived as follows:

(9) $$\eta_{t}/\eta_{0}=exp(exp(30.757-12904/T)t)$$

Subsequently, combining Equations (5) and (9) in the final correlation among $\eta_{t}$, *T*, and *t*, serving as the chemical rheological model for the epoxy resin, specifically, gives

(10) $$\eta_{t}=exp(exp(30.757-12904/T)t)\times (7.029\times 10^{-7}exp(6480.8/T))$$

That was

(11) $$\eta_{t}=7.029\times 10^{-7}exp[exp(30.757-12904/T)t+(6480.8/T)]$$

As presented in Figure 1f, the experimental data agreed well with the curves predicted by the chemical rheological model, demonstrating a high level of consistency and indicating good predictive accuracy. The developed chemical rheological model for the epoxy resin provides a theoretical basis for defining the processing temperature window and constraining the processing time during prepreg fabrication.

### 2.2. Curing Temperature Range of Epoxy Resin

**Figure 2:**
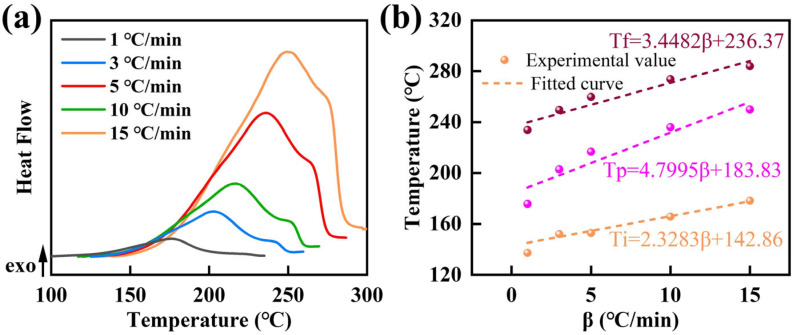
(**a**) DSC curves of epoxy resin at different heating rates and (**b**) the *T-β* curves for epoxy resin.

The curing behavior of epoxy resin at different heating rates was characterized by non-isothermal DSC. Figure 2 showed the DSC curves and characteristic temperatures at different heating rates. The DSC traces exhibited two exothermic peaks as exhibited in Figure 2a, and the exothermic process was completed below 300 °C, indicating that the resin can be fully cured within this temperature range.

With increasing heating rate, the exothermic peak temperature shifted toward higher temperatures, accompanied by an increase in the reaction enthalpy. Meanwhile, the initial temperature (*T_i_*), peak temperature (*T_p_*) and final temperature (*T_f_*) of the exothermic peak of the exothermic event all increased [28,29]. This behavior could be attributed to the reduced residence time at lower temperatures at higher heating rates, which delayed the progression of the curing reaction and consequently shifted the characteristic temperatures to higher values [30,31].

(12) T=A+Bβ

The *T_i_*, *T_p_*, and *T_f_* at different heating rates were listed in Table 2. Accordingly, the *T* and *β* relationships were established by plotting the characteristic temperatures *T_i_*, *T_p_*, and *T_f_* as functions of the heating rate (*β*) in Figure 2b. The characteristic temperature (*T_i_*, *T_p_*, and *T_f_*) varies linearly with the heating rate *β* [28,32], as expressed by Equation (12). And three curves of *T_i_-β*, *T_p_-β* and *T_f_-β* were fitted based on the heating rate and characteristic temperature, respectively. Each curve was extrapolated to *β* = 0 to obtain the corresponding intercept, which defined the gelation temperature (*T_i_*), curing temperature (*T_p_*), and post-curing temperature (*T_f_*) of the epoxy resin, respectively. The calculated values of *T_i_*, *T_p_*, and *T_f_* were 142.86 °C, 183.83 °C, and 236.37 °C at *β* = 0, respectively, as summarized in Table 3. These results indicated that the curing temperature window of the resin spanned 142.86–236.37 °C.

### 2.3. Curing Kinetics Equation of nth-Order Reaction for Epoxy Resin

For curing reactions of thermosetting resins, the kinetics is most described using an *n*th-order reaction model [20,21,33], expressed as follows:

(13) $$\frac{da}{dt}={k(1-a)}^{n}$$

where α denotes the degree of conversion, *t* is the reaction time (s), *k* is the curing rate constant, and *n* is the reaction order.

Under isothermal conditions, the temperature *T* dependence of the rate constant *k* is commonly described by the Arrhenius equation, given as follows:

(14) $$k=Aexp(-\frac{E_{a}}{RT})$$

where *T* represents the absolute temperature.

By combining Equations (13) and (14), the *n*th-order curing kinetics equation was obtained as follows:

(15) $$\;\frac{da}{dt}={Aexp(-\frac{E_{a}}{RT})(1-a)}^{n}$$

Subsequently, the model parameters $E_{a}$, *n* and *A* were determined. The activation energy $E_{a}\;$ was evaluated using three methods, as summarized below.

The Kissinger equation [28,32] was given as follows:

(16) $$\ln\frac{\beta }{T_{p}^{2}}=\ln(\frac{AR}{E_{a}})-\frac{E_{a}}{R}\times \frac{1}{T_{P}}$$

The Ozawa equation [34,35] was written as follows:

(17) $$\frac{ln\beta }{\frac{1}{T_{P}}}=-\frac{{1.052E}_{a}}{R}$$

The Straink equation [34,36] was expressed as:

(18) $$ln\frac{\beta }{{Ta}^{1.92}}=C-1.0008\frac{E_{a}}{RTa}$$

In the previous equations, *A* is the pre-exponential factor (S^−1^), *R* is the universal gas constant (8.3145 J/(mol·K)), *Tp* is the peak temperature (K), and *Ta* denotes the temperature corresponding to a given degree of cure.

**Figure 3:**
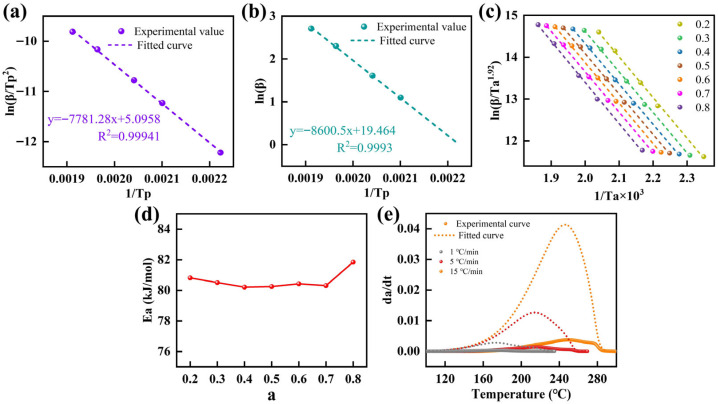
Determination of kinetic parameters from DSC data. (**a**) $\ln\frac{\beta }{T_{p}^{2}}$ versus $\frac{1}{T_{P}}$. (**b**) $\ln\frac{\beta }{T_{p}^{2}}$ versus $\frac{1}{T_{P}}.$ (**c**) $ln\frac{\beta }{{Ta}^{1.92}}$ versus $\frac{1}{Ta}$. (**d**) $E_{a}$ as a function of degree of cure in the range of 0.2 ≤ *a* ≤ 0.8. (**e**) Comparison between experimental and model-predicted *da/dt* as a function of temperature.

According to Equation (16), Figure 3a was constructed, and *Ea* was obtained from the slope of the fitted line as 64,693.562 J·mol^−1^. Furthermore, Figure 3b was generated based on Equation (17), and linear fitting of the corresponding plot yielded $E_{a}$ = 67,970.111 J·mol^−1^ from the slope. Following Equation (18), the characteristic curing temperatures at different heating rates were used to establish the corresponding plot in Figure 3c. The *Ea* as a function of conversion *a* was then determined from the fitted slope. The resulting *Ea* values in the range of 0.2 ≤ *a* ≤ 0.8 were shown in Figure 3d, with an average of 80,620.616 J·mol^−1^. The individual values reflected methodological differences but fall within a comparable range; thus, an averaged $E_{a}$ value of 71,094.763 J·mol^−1^, derived from the three methods, was reported only as a reference.

Subsequently, the reaction order *n* was determined using the Crane method [37,38]:

(19) $$\frac{dln\beta }{d(\frac{1}{T_{P}})}=-\frac{E_{a}}{nR}$$

Based on the Crane method in Equation (19) and the slope of the fitted line in Figure 3b, the reaction order was calculated by substituting the obtained $E_{a}$, giving *n* = 0.9943. The pre-exponential factor *A* was further calculated from the intercept of the Kissinger plot (Equation (16)) in Figure 3a, yielding *A* = 1.397 × 10^6^ S^−1^. By inserting *Ea*, *n*, and *A* into Equation (15), the *n*th-order curing kinetics equation for the epoxy resin was obtained as follows:

(20) $$\frac{da}{dt}={1.397\times 10^{6}exp(-8600.5/T)(1-a)}^{0.9943}$$

The *n*th-order kinetic equation was used to predict *dα/dt* at heating rates of 1 °C/min, 5 °C/min and 15 °C/min, and the predictions were compared with the corresponding experimental results, as exhibited in Figure 3e. A pronounced discrepancy was observed between the calculated and measured *dα/dt* profiles, indicating that the *n*th-order model was not suitable for this epoxy resin.

### 2.4. Curing Kinetic Equation of Autocatalytic Reaction of Epoxy Resin

**Figure 4:**
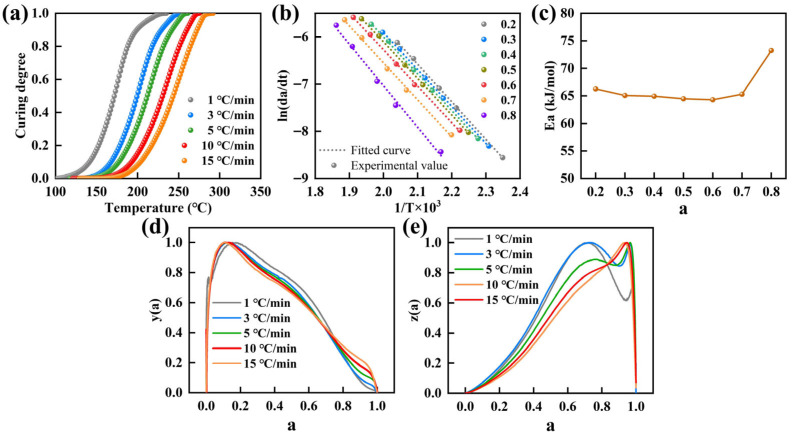
(**a**) The relationship between the curing degree *a* and temperature *T*. (**b**) Linear fitting curves of ln(*da*/*dt*) and 1/*T,* and (**c**) *Ea* within the range of 0.2 ≤ *a* ≤ 0.8. The relationship between (**d**) *y*(*a*), (**e**) *z*(*a*), and *a* at different heating rates.

To determine whether the curing process of the epoxy resin exhibited autocatalytic characteristics, the data in Figure 2a were converted into the curing degree *a* versus temperature *T* curves in Figure 4a. The resulting curves were distinctly S-shaped, indicating pronounced autocatalytic behavior during curing of the epoxy resin [39,40].

Consequently, a two-parameter autocatalytic kinetic model was adopted to describe the curing kinetics of the epoxy resin, and the Málek conversion-rate formulation was introduced for the analysis [30,41]:

(21) $$\frac{da}{dt}=Aexp(-\frac{E_{a}}{RT})f(a)$$

where $f\left(a\right)=a^{m}{\left(1-a\right)}^{n}$, *m* is the reaction order associated with the autocatalytic term, and *n* is the reaction order related to the curing-agent contribution. Thus, the formula for the autocatalytic model was as follows:

(22) $$\frac{da}{dt}=Aexp(-\frac{E_{a}}{RT})a^{m}{\left(1-a\right)}^{n}$$

Applying a logarithm transformation to Equation (22) gave

(23) $$\ln\frac{da}{dt}=\ln A+m\ln a+n\ln\left(1-a\right)-\frac{E_{a}}{RT}$$

Based on Equation (22), ln(*da/dt*) was plotted against 1/*T* at degrees of cure *a* = 0.2–0.8, yielding a linear relationship in Figure 4b. The slope of the fitted line corresponded to $-E_{a}$*/R*, from which the $E_{a}$ was obtained. The resulting $E_{a}$ values as a function of *a* were shown in Figure 4c. Averaging *Ea* over *a* = 0.2–0.8 gave $E_{a}$ = 66,221.604 J·mol^−1^.

To further verify whether the curing reaction followed an autocatalytic mechanism, the *y*(*a*) and *z*(*a*) functions proposed by Málek were employed [30,41]. These functions were auxiliary kinetic functions derived from the normalized reaction rate and were commonly used to identify the underlying reaction mechanism of thermosetting systems. The *y*(*a*) function highlighted the contribution of the autocatalytic term at different degrees of conversion, whereas the *z*(*a*) function reflected the combined influence of temperature and reaction progress. The relative positions of the maxima of *y*(*a*) and *z*(*a*) provided qualitative criteria for distinguishing between different kinetic models. In particular, the conditions of 0 < $a_{M}$ < $a_{p}^{\infty }$ and $a_{p}^{\infty }$ ≠ 0.632 were indicative of autocatalytic reaction behavior. The formulas for *y*(*a*) and *z*(*a*) were defined as follows:

(24) $$y\left(a\right)=(\frac{da}{dt})e^{x}$$

(25) $$z\left(a\right)=\pi (x)(\frac{da}{dt})\frac{T}{\beta }$$

where $x=E_{a}/RT$ and *π(x)* denoted the fourth-order rational approximation of the temperature integral proposed by Semun-Yang [42], given by:

(26) $$\pi (x)=\frac{e^{-x}}{x}\frac{x^{3}+{18x}^{2}+88x+96}{x^{4}+{20x}^{3}+{120x}^{2}+240x+120}$$

By substituting the $E_{a}$ obtained in Figure 4c into the expressions for *y*(*a*) and *z*(*a*), the corresponding *y*(*a*)–*a* and *z*(*a*)–*a* relationships were determined. After normalization, the resulting curves were presented in Figure 4d,e. The values at which *y*(*a*) and *z*(*a*) reached their maxima were denoted as $a_{M}$ and $a_{p}^{\infty }$, respectively, and the corresponding values at different heating rates were listed in Table 4. As summarized in Table 4, the criteria 0 < $a_{M}$ < $a_{p}^{\infty }$ and $a_{p}^{\infty }$ ≠ 0.632 were satisfied, confirming that the curing reaction of the epoxy resin followed an autocatalytic (*m*, *n)* kinetic model [39,43].

Equation (23) was treated as a function of the *a*, and fitted by nonlinear regression with iterative optimization to determine *lnA* and the parameters *m* and *n* for each heating rate. The resulting values of *m*, *n* and *A*, along with their mean values, are summarized in Table 5. For clarity, the kinetic parameters of the autocatalytic model, namely A, $E_{a}$, m, and n, were summarized in Table 6.

Substituting the mean *m*, *n* and *A* values into Equation (22) yielded the corresponding curing kinetics equation, as follows:

(27) $$\frac{da}{dt}=42597.69843exp(-\frac{7965.07143}{T})a^{0.277702}{\left(1-a\right)}^{0.883772}$$

**Figure 5:**
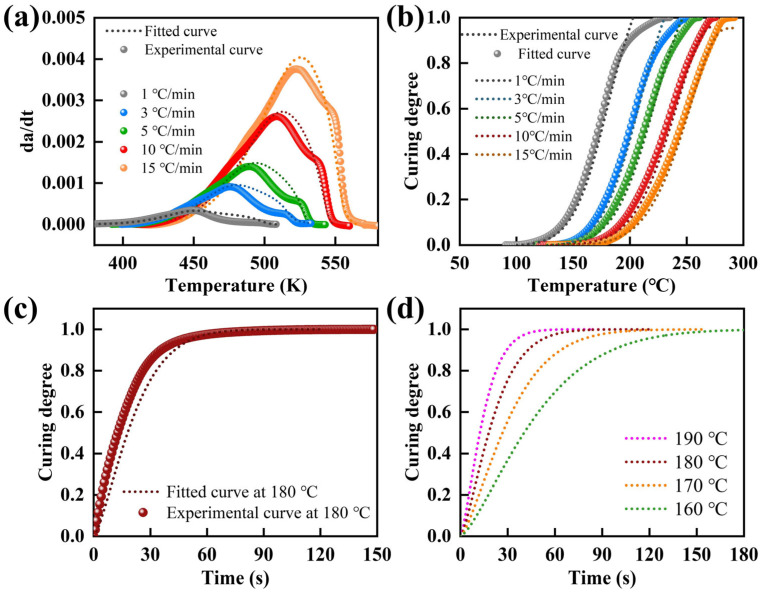
Comparison between experimental and model-predicted (**a**) *da/dt* and (**b**) curing degree at different temperatures. (**c**) The curing degree predicted by the model was verified by an isothermal experiment at 180 °C. (**d**) Model-predicted evolution of curing degree with time at various temperatures.

Subsequently, Equation (27) was used to calculate the *da/dt-T* and *a-T* profiles at different heating rates. The calculated curves were compared with the corresponding experimental data, as presented in Figure 5a,b. Good agreement was observed for both the *da/dt-T* and *a-T* relationships, suggesting that the autocatalytic model could reliably describe and predict the curing behavior of the epoxy resin.

The results in Figure 5a,b validated the predictive capability of the autocatalytic model under non-isothermal conditions. To further assess its applicability under isothermal curing, the relationship of *a* and *t* at 180 °C was calculated using Equation (27), as presented in Figure 5c. In parallel, isothermal DSC measurements were performed for epoxy resin at 180 °C to obtain the experimental *a–t* curve. Comparison between the experimental data and Equation (27) predictions showed good agreement, indicating that Equation (27) could be used to estimate the curing time under isothermal conditions. Furthermore, the autocatalytic kinetic model was employed to predict the evolution of *a* with time at 160 °C, 170 °C, 180 °C, and 190 °C under isothermal conditions (Figure 5d), enabling estimation of the time required to reach complete cure at each temperature. As expected, the curing time decreased markedly with increasing isothermal temperature. The predictions provide a quantitative basis for designing and controlling the curing and forming schedules of the resin and its composites.

## 3. Experimental Sections

### 3.1. Materials

**Figure 6:**
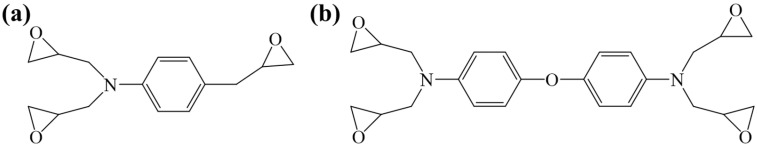
Chemical structures of the (**a**) trifunctional and (**b**) tetrafunctional epoxy resins used in the ER220 formulation.

A high-performance multifunctional epoxy resin (ER220) was purchased from Suzhou Shanmuxi New Materials Co., Ltd. (Suzhou, China). The formulation consisted primarily of a tetrafunctional epoxy resin and a trifunctional epoxy resin, with poly (ether sulfone) (PES) as a toughening agent, 4,4′-diaminodiphenyl sulfone as the curing agent, and dicyandiamide as an accelerator. The epoxy equivalent weight, calculated based on the epoxy-containing components, was approximately 102 g·eq^−1^, and the non-volatile content was below 1%. The chemical structures of the tetrafunctional and trifunctional epoxy resins are shown in Figure 6.

### 3.2. Characterizations

A rotational rheometer (Kinexus PRO+, NETZSCH-Gerätebau GmbH, Selb, Germany) was employed to characterize the rheological behavior of the epoxy resin. Viscosity–temperature profiles were recorded from 50 °C to 180 °C at heating rates of 1 °C/min and 3 °C/min to determine the temperature window relevant to prepreg processing. In this basis, isothermal rheological tests were performed at 75 °C, 82.5 °C, 90 °C, and 97.5 °C for 180 min, respectively. The curing kinetics of the epoxy resin were investigated under a nitrogen atmosphere using a DSC 214 differential scanning calorimeter (NETZSCH-Gerätebau GmbH, Selb, Germany) with aluminum crucibles. Measurements were performed from 50 °C to 300 °C at heating rates of 1 °C/min, 3 °C/min, 5 °C/min, 10 °C/min, and 15 °C/min. Moreover, an isothermal test was conducted at 180 °C.

## 4. Conclusions

In this work, the rheological behavior and curing kinetics of a high-performance epoxy resin were systematically characterized, modeled, and validated. For rheology, a rotational rheometer was used to obtain the dynamic viscosity–temperature profiles, based on which isothermal rheological measurements at selected temperatures were conducted and a corresponding chemorheological model was established. The model predictions agreed well with the experimental data, demonstrating that the proposed model can effectively capture the viscosity evolution of the resin within the processing-relevant temperature window. For cure kinetics, the non-isothermal curing behavior was investigated by DSC at multiple heating rates. An *n*th-order kinetic model was first assessed based on the DSC data but was found inadequate to describe the curing behavior of this epoxy resin. Subsequently, the conversion characteristics were examined to assess whether a two-parameter autocatalytic mechanism was applicable. An autocatalytic kinetic model was subsequently developed to describe the curing process, and its reliability and predictive accuracy were confirmed by the good agreement between model predictions and both non-isothermal and isothermal experimental data. Overall, the developed rheological model provides guidance for resin processing and prepreg fabrication, whereas the curing kinetics model offers a quantitative basis for designing curing and forming schedules for the resin and its composites. The established characterization–modeling–validation method provides a general framework for modeling the rheological and curing kinetics of epoxy resins.

## Figures and Tables

**Table 1:** The *n* values of the epoxy resin system at different temperatures.

T/°C	75	82.5	90	97.5
*n*	0.0018	0.0038	0.0097	0.016

**Table 2:** Characteristic temperatures of epoxy resins at different heating rates.

Heating Rate/(°C/min)	*T_i_*/°C	*T_p_*/°C	*T_f_*/°C	*ΔT*/°C
1	137.30	175.72	233.87	202.63
3	152.04	202.93	249.59	201.84
5	152.89	216.78	259.82	201.15
10	165.78	236.01	273.79	187.83
15	178.16	249.91	284.02	181.82

**Table 3:** Characteristic temperature at *β* = 0 °C/min calculated based on *T-β* extrapolation method.

Heating Rate/(°C/min)	*T_i_*/°C	*T_p_*/°C	*T_f_*/°C	*ΔT*/°C
0	142.86	183.83	236.37	78.23

**Table 4:** The values of $a_{M}$ and $a_{p}^{\infty }$ obtained by DSC corresponding to different heating rates.

Heating Rate/(°C/min)	1	3	5	10	15
$a_{M}$	0.1546	0.1289	0.1149	0.1102	0.1069
$a_{p}^{\infty }$	0.7176	0.7297	0.9666	0.9341	0.9464

**Table 5:** Curing kinetic parameters of autocatalytic model of epoxy resin.

Heating Rate/(°C/min)	*m*	*n*	*A*
1	0.10101	0.99506	31,318.3667
3	0.29631	1.02972	45,588.46486
5	0.28176	0.80019	39,386.16927
10	0.39647	0.82262	50,904.16366
15	0.31296	0.77102	45,791.32767
Mean value	0.277702	0.883722	42,597.69843

**Table 6:** Autocatalytic kinetic parameters of the epoxy resin.

A	$E_{a}$	*m*	*n*
42,597.69843	66,221.604 J·mol^−1^	0.277702	0.883722

## Data Availability

Data will be made available on request.
